# “A cup of tea with our CBD agent … ”: community provision of injectable contraceptives in Kenya is safe and feasible

**DOI:** 10.9745/GHSP-D-13-00040

**Published:** 2013-09-30

**Authors:** Alice Auma Olawo, Issak Bashir, Marsden Solomon, John Stanback, Baker Maggwa Ndugga, Isaac Malonza

**Affiliations:** a FHI 360, Nairobi, Kenya; b Ministry of Health, Nairobi, Kenya; c FHI 360, Research Triangle Park, NC, USA; d Jhpiego/Kenya, Nairobi, Kenya

## Abstract

**Background:**

In rural areas of Kenya, where the majority of Kenya′s population lives, contraceptive use remains low compared with that in urban areas (37% vs. 47%). Inadequate access to family planning services in rural areas is partly due to fewer health facilities and the shortage of health care workers. Community-based access to injectable contraceptives can improve access for rural populations and expand the range of contraceptive methods available. Our pilot project sought to generate local evidence on safety, feasibility, and acceptability of the provision of injectable depot medroxyprogesterone acetate (DMPA) by community health workers (CHWs).

**Design:**

We trained 31 CHWs in Tharaka District to provide injectable DMPA in addition to pills and condoms. Data were collected on family planning clients served by CHWs in Tharaka District as well as those who received services from health facilities from August 2009 to September 2010. Service statistics were collected from 3 pilot health facilities in the CHW service catchment area.

**Results:**

In the 12-month study period, CHWs reached 1,210 women with family planning services including referrals for long-acting and permanent methods. Family planning use in the pilot sites for all methods increased an estimated fivefold, from 9% in facilities to 46% when facilities and CHWs were combined (32% for CHWs and 14% for facilities). The majority (69%) of clients served by CHWs chose DMPA. No client reported any signs of infection at the injection site nor did any CHW report needlestick injuries or other adverse events. The re-injection rate was 68% at the third visit, which compares favorably with other DMPA continuation studies. Two main reasons given for discontinuing were change of residence and temporary separation from spouse.

**Conclusion:**

Community-based provision of DMPA along with other contraceptive methods increased the use of family planning and improved method choice during the study period. Injectable contraception provided by trained CHWs is a safe, acceptable, and feasible service delivery option in Kenya.

## Abstract (precis)

Community health workers can safely provide the injectable DMPA when appropriately trained and supervised. We also found a fivefold increase in contraceptive uptake—a finding that builds on evidence from other countries for supportive policy change.

## BACKGROUND

Kenya made significant gains in key reproductive health indicators between the late 1970s and the late 1990s, with the total fertility rate (TFR) declining from 8 to 5 births per woman and the contraceptive prevalence rate (CPR) increasing from 7% to 39% among currently married women. However, Kenya experienced stagnation in the CPR and little change in fertility rates in the period 2000 to 2010.

Contraceptive use remains low in rural areas (37%) compared with urban areas (47%). Use of pills in the urban areas is almost double that in rural areas (11% versus 6%), as it is for the intrauterine contraceptive device (IUD) (2.9% versus 1.2%). Injectable contraceptive use is at 23.5% in urban areas and 21% in rural areas, while implant use is 2.7% and 1.7%, respectively.^1^

These disparities could partly be a result of access. A majority (70%) of Kenya's population lives in rural areas where many people do not have adequate access to family planning services. Many live at considerable distance from the nearest health facility; when they do have reasonable access to a facility, services are often limited due to health care worker shortages.

Approaches such as community-based access to injectable contraceptives (CBA2I) can improve access for this population and expand the range of contraceptive methods available. CBA2I has been practiced in Asia since the 1970s, and more recently in Latin America and Africa. It is recognized as a safe, acceptable, and effective means to increase access to family planning method choices and to promote healthy birth spacing among underserved populations with poor access to health facilities.^2^ The World Health Organization (WHO) now recommends optimizing health worker roles through task sharing, including CBA2I to improve access to key maternal and newborn health interventions.^3^

Community health workers (CHW) now provide injectable contraceptives in more than 10 African countries. Madagascar, Nigeria, Rwanda, and Uganda have revised their national reproductive health policy guidelines to allow CHWs to provide injectable contraceptives, and Malawi's and Senegal's Ministries of Health have agreed to support national provision of injectables by 1 cadre of CHWs.

In Kenya's rural areas, where contraceptive use remains low, community-based injectable contraceptives could boost access and increase method choice.

With the realization that an improvement in reproductive health indicators can only be accomplished by involving the community, Kenya is currently focusing on building the capacity of the community to provide community-based health services. A recent study assessing the current family planning practices at the community level indicated that CHWs want to be trained to provide injectable contraceptives in order to provide their clients with a wider range of family planning services.^4^

Kenya's current strategy for delivering community-based health services allows community health workers to provide both family planning information and selected services.^5^ These include counseling on all family planning methods and provision of methods such as condoms, pills, the Lactational Amenorrhea Method, and the Standard Days Method®.^6^ However, the family planning guidelines do not allow community health workers to provide injectable contraceptives despite the method's clear popularity.

A CBA2I advocacy campaign began in Kenya in 2006 with sensitization efforts targeting key stakeholders in the medical field, such as medical professional associations and Ministry of Health (MOH) stakeholders at the provincial level. We conducted a study tour to Uganda in March 2007 to enable Kenyan stakeholders to learn from that country's experience. Stakeholders recommended a pilot project to determine CBA2I safety, feasibility, and acceptability in the Kenyan context as well as formation of an advisory committee to oversee implementation of the pilot. A project advisory committee (PAC) led by the MOH's Division of Reproductive Health (DRH) and comprising representatives from other organizations, including FHI 360, Jhpiego and the U.S. Agency for International Development's (USAID) AIDS, Population and Health Integrated Assistance (APHIA II) Eastern program, among others, was formed. The PAC identified Tharaka District as a suitable site for the pilot study for a number of reasons, including existence of an active community-based distribution program, willingness of the District Health Management Team (DHMT) to support the pilot, and poor access to health facilities. The Tharaka DHMT became an automatic member of the PAC once the site was selected. The PAC identified 3 health facilities to which CHWs would be attached, namely, Kibung'a Sub District Hospital, Kanyuru Health Center, and Rukenya Dispensary.

The main purpose of the pilot was to generate local evidence to determine the safety, feasibility, and acceptability of community-based distribution of injectable depot medroxyprogesterone acetate (DMPA) by community health workers in Kenya. Despite existing evidence from other countries, such as Uganda, the Kenya MOH and stakeholders felt it was important to conduct a pilot to establish whether the same experiences could be replicated in Kenya.

## METHODS

### Pre-Implementation Procedures

The pilot study was implemented between August 2009 and September 2010. The CHWs completed training at the end of August 2009, and the majority started serving clients the following month, September 2009. The indicators for the pilot project were:

- Total number of family planning users (both clinic and CHW clients) in the intervention area prior to and during the pilot
- Number of returning DMPA clients served in the clinics to which CHWs were attached
- 12-month continuation rates for CHW clients using DMPA (as measured by the proportion of clients receiving an injection at 9 months)

Kenya stakeholders regard referrals as a key component of CHW work. They were therefore keen on CHWs not just providing the methods they had been trained on but also making referrals for other methods.

Other activities undertaken during implementation of the CBA2I initiative included identification of CHWs, development of training materials, training of CHWs, and monitoring and support supervision.

### Community Health Worker Identification and Training

Representatives of the PAC led by the DRH, and in collaboration with local government officials, selected and trained 31 CHWs to provide DMPA in addition to the pills and condoms they were already providing. Selection criteria included residence in the study site, respect in the community, and at least 7 years of primary education (which was deemed the minimum amount of educational attainment necessary to facilitate comprehension of their tasks and the job aids provided). They also had to have previous CHW experience with provision of family planning services (counseling, condoms, and pills) at the community level.

Extensive training prepared 31 community health workers to provide injectable DMPA along with other methods and to make effective referrals.

The 31 CHWs underwent classroom and practical training for 3 weeks. Trainers included MOH trainers from the national, provincial, and district levels as well other PAC members. Classroom training, most of which was done in group-work format to enhance understanding, involved a review of all family planning methods. Rumors and misconceptions related to family planning were also discussed. A considerable amount of time was spent equipping the CHWs with counseling skills through role-play techniques. In addition, CHWs were also oriented to family planning checklists for ruling out pregnancy, providing contraceptive pills, and providing DMPA. The CHWs also learned about infection-prevention procedures, safe-injection techniques, appropriate record keeping, and effective referrals.

During practical training, substantial time was spent practicing injection techniques using tomatoes and oranges. The CHWs were divided into groups of 3 and each assigned a mentor to ensure they learned proper injection technique. Other teaching methods included, but were not limited to, demonstrations and return demonstrations (trainees' successful repetition of a technique previously demonstrated to them), case studies, lectures, and brainstorming. Materials used for training were a reference manual, participant manual, trainer's manuals, CHW job aids, re-injection calendars, and record-keeping tools.

Classroom and practical training were followed by a 2-week clinical practicum to allow CHWs to provide family planning services to clinic clients under the supervision of a nurse. The CHWs had to achieve pre-determined competence standards, including correct administration of at least 5 injections, before being certified to administer injections on their own.

### Monitoring and Support Supervision

Two registered nurses supervised the CHWs; 1 nurse was in charge of 1 of the catchment area health facilities (4 CHWs) and the other nurse of the 2 remaining catchment areas (31 CHWs). The supervisors made at least 1 home visit to each of the CHWs but, due to time constraints, they scheduled supervision once per week at the health facility, where 2 CHWs could spend a whole day providing family planning services under supervision. When possible, the supervisors observed CHW service provision during the home visits. They also discussed with the CHWs any challenges faced during service provision and worked together with CHWs on plans to address these. The supervisors used a checklist to guide their discussion and recorded their observations.

The CHWs obtained their commodities and other supplies from the health facilities to which they were attached. Commodities and supplies for the pilot were made available through the normal district ordering system. The CHWs were trained to use a monthly tracking tool to order commodities and other supplies.

Apart from onsite supervision provided by the CHW supervisors described above, we undertook additional monitoring and support supervision on a monthly basis, later reduced to 1 visit every 2 months as the CHWs gained more experience in service provision and record keeping. We reviewed client records, the referral records, and the monthly tracking tool. We also compiled data on the project indicators during the visits. In addition, we compiled service delivery data from the 3 health facilities to which the CHWs were affiliated. The project team also took the opportunity to refresh CHW knowledge by asking questions about what was taught during training.

### Data Collection Procedures

Recruitment of participants was continuous throughout the 12-month pilot period. Clients receiving DMPA injections were followed at 3, 6, and 9 months to monitor continuation rates. Process monitoring data were obtained from client tracking cards, referral cards, and family planning service statistics from the 3 facilities to which CHWs were attached.

The 3 indicators that formed the basis of data collection for the pilot project were:

- Total number of family planning users (both clinic and CHW clients) in the intervention area prior to and during the pilot
- Number of returning DMPA clients served in the clinics to which CHWs were attached
- 12-month continuation rates for CHW clients using DMPA (as measured by the proportion of clients receiving an injection at 9 months)

Data on the above indicators were collected by CHWs. The CHWs completed a tracking card for each client whom they served. Information recorded on the client tracking card included age of the client, method provided, type of user (whether new or returning), and any referral made. One tracking card per client was used to record all visits made within the pilot period. Data extracted from the client tracking card informed the first and the third indicators (total number of family planning users for the CHWs and 12–month re-injection and discontinuation rates). Service statistics from family planning registers at the 3 facilities to which the CHWs were attached contributed to the measurement of the first and second indicators (total number of family planning users for the health facilities and number of returning DMPA clients served in clinics to which CHWs were attached).

All data were entered, cleaned, and analyzed using Excel, version 97–2003. We did not seek ethical approval since the data to be collected came from process monitoring and not direct contact with clients.

## RESULTS

### Family Planning Users Served By Community Health Workers

Table 1 shows the number of family planning clients reached by CHWs by method and type of client. The pilot project reached 1,210 women in rural Tharaka with a range of family planning services. Two-thirds (69%) of these women either initiated or continued using DMPA through CHWs, and the rest were pill or condom acceptors or were referred for clinical methods. Of DMPA acceptors, 14% were new to family planning and 12% had switched to DMPA from oral contraceptive pills or other methods. CHWs initiated new DMPA clients using the appropriate family planning checklist. About three-quarters (74%) of DMPA clients who had previously received DMPA from a clinic opted to receive it from CHWs.

DPMA was the method of choice for 69% of women reached by CHWs.

**Table 1.** Family Planning Services Provided by CHWs, by Contraceptive Method and Type of Client (August 2009–September 2010)

**Method**	**No. of clients**	**% of total clients (N = 1210)**	**% of client type using method**
**Depo-Provera (DMPA)**			
Former clinic clients	614	51	74
New to family planning	118	10	14
New to DMPA	100	8	12
**Subtotals**	**832**	**69**	**100**
**Pills** (including emergency contraceptives)			
New users	53	4	36
Returning users	96	8	64
**Subtotals**	**149**	**12**	**100**
**Condoms**			
New users	102	8	52
Returning users	93	8	48
**Subtotals**	**195**^a^	**16**	**100**
**Referrals**			
BTL	9	1	25
IUD	15	2	35
Implants	5	1	25
Side effects	2	1	6
Other	3	1	6
**Subtotals**	**34**	**3**^b^	**97**^b^
**TOTAL**	**1210**	**100**	

a 56% (109) of the condom users were dual method users.

b Percentage inexact due to rounding

Abbreviations: BTL, bilateral tubal ligation; CHW, community health worker; DMPA, depot medroxyprogesterone acetate; IUD, intrauterine contraceptive device.

The results showed that CHWs made 34 referrals. Of these, 15% were for implants, 44% for IUDs, and 27% for female sterilization. A few clients (6%) were referred for side effects and other procedures (9%) such as pregnancy testing, cancer screening, and voluntary counseling and testing (VCT). The clients were referred to various health facilities, including the 3 facilities to which CHWs were attached: Tharaka District Hospital and Marie Stopes mobile outreach services.

Overall family planning use in the catchment area increased fivefold, from 9% at baseline to 46% when CHW provision (32%) was added to facility-based services (14%). (Overall family planning use was calculated using population census figures of women of reproductive age in the 3 pilot locations as the denominator and clinic service statistics and CHW records as the numerator.) At baseline, DMPA use in catchment areas was 6%, but it increased to 38% after adding CHW provision of this method (25% for CHWs, 13% for facilities).

When CHW provision was added to facility-based services, overall family planning use in the catchment area increased fivefold, from 9% to 46%.

### Family Planning Users Served in Clinics

Table 2 presents the number of clinic clients accessing various family planning services in Kanyuru, Rukenya, and Kibung'a dispensaries 3 months before the study period and during the first 3 and last 3 months of CHW provision of DMPA. These facilities experienced an increase from 166 to 263 DMPA clients (new and returning) (58% increase) in the first 3 months of the pilot and a further increase to 341 clients (30% increase) at the end of the study period (6 months later).

**Table 2.** Clinic-Based Family Planning Services Provided Before and During the Pilot Intervention

**Method provided**	**3 months before intervention (Jun–Aug 2009)**	**First 3 months of intervention (Sep–Nov 2009)**	**Last 3 months of intervention (May–Jul 2010)**	**Total**
**DMPA**	166	263	341	**770**
**COCs**	59	12	29	**100**
**POPs**	4	1	3	**8**
**Implants**	4	0	0	**4**
**IUD**	0	3	0	**3**

Abbreviations: COC, combined oral contraceptives; DMPA, depot medroxyprogesterone acetate; IUD, intrauterine contraceptive device; POP, progestin-only pills.

It appears that contraceptive pills may have become slightly less popular compared with DMPA, as evidenced by the decrease in pill clients (new and returning) over the course of the pilot period.

### Re-Injection

Table 3 shows the proportion of eligible clients who received a re-injection from a CHW. The re-injection rates were 89%, 81%, and 68% at 3, 6, and 9 months, respectively. The 12-month continuation rate of 68% compares favorably with other studies of DMPA continuation.^7^

**Table 3.** Re-Injection Rate Among CHW DMPA Clients

	**Eligible Clients Receiving Re-Injections**			**Reported related needlestick injuries (n = 2453^a^)**
	**1^st^ re-injection (at 3 months) (n = 761)**	**2^nd^ re-injection (at 6 months) (n = 672)**	**3^rd^ re-injection (at 9 months) (n = 508)**	
**Tharaka pilot sites** (31 CHWs)	89%	81%	68%	0% (0)

Percentages calculated among those clients who were eligible for a second, third, or fourth injection

a Total number of DMPA injections provided by all CHWs during the pilot period

Abbreviations: CHW, community health worker; DMPA, depot medroxyprogesterone acetate.

### Reasons for Discontinuation

As a secondary objective, we sought to determine reasons for discontinuation of DMPA services. The Kenya Demographic and Health Survey mentions side effects and desire to become pregnant as the 2 main reasons why clients discontinue use of DMPA.^8^ In the pilot, the 2 main reasons given for discontinuing were changing residence and temporary separation from spouse. Other reasons included the desire to get pregnant and side effects. Some clients decided to discontinue DMPA use because of amenorrhea and/or desire to switch to another family planning method. Other clients chose not to receive their re-injection because their husband was away, and they felt they did not need contraception. A few clients who expressed an intention to continue with DMPA missed their re-injections due to flooded roads or other natural phenomena that prevented travel to the CHW's home. In the majority of these cases, CHWs were not able to reach clients to establish reasons for discontinuation.

## DISCUSSION

### Safe, Feasible, and Acceptable

The objective of the pilot study was to determine the safety, feasibility, and acceptability of CBA2I in Kenya. Safety was addressed by the occurrence and frequency of adverse events reported by the CHWs. Such adverse events included abscesses or needlesticks. Of the 2,453 injections provided by the CHWs, no cases of injection site infection or needlesticks were reported—an indication that the CHWs provided the services safely.

To address feasibility, the pilot study was implemented within existing MOH structures. Tharaka DHMT successfully implemented the pilot by putting in place supervision and other support systems including monitoring visits and provision of resupply. The pilot was deemed feasible because the DHMT provided full support and took the lead in all monitoring activities. To date, the DHMT has continued to support the CHWs and has indicated willingness to scale up to other areas once scale-up implementation guidelines are put in place.

The practice of CHW provision of DMPA was also found to be acceptable, as evidenced by the number of clients who were reached by the CHWs (N = 1,210). About three-quarters (74%) of DMPA clients who had previously received DMPA from a clinic opted to switch to CHWs. This has the benefit of reducing the client burden on busy nurses and midwives, allowing them to better use their specialized clinical skills, while allowing a lower cadre to perform a more routine task.

While there is no single standard measure for the quality of community-based delivery of injectables, re-injection rate is a proxy measure for quality because it suggests a degree of client satisfaction with the method and services received. The 12-month continuation rate of 68% (measured by acceptance of a fourth injection at 9 months) seen in the study compares favorably with other studies of DMPA continuation. This reflects well on the ability and performance of CHWs as well as client satisfaction with the method and services. Since the CHWs were based in the community, they were able to follow up with the clients who forgot their re-injection dates, thus helping to enhance DMPA continuation.

Since the CHWs were based in the community, they were able to follow up with the clients who forgot their re-injection dates.

Another indicator of acceptability is the enthusiasm shown by clients of the CHWs, as illustrated by anecdotal information gathered by the monitoring team during their field visits:

The hospital is so far away; we waste time there and we have to carry our babies along, leaving work unattended.

We can visit our CBD at any time, even on our way from the farm or market.

These days, we take a cup of tea with our CBD agent as we discuss real family issues (sexuality, child health, STI, etc.).

### The Spill-Over Effect

During the pilot, CHWs referred clients to health facilities for longer-acting and reversible methods, and there appears to be a “spill-over effect” to non-family planning services, as seen in the net increase in deliveries by skilled birth attendants and immunization rates, as well as an overall increase in the uptake of family planning in the district hospital. The spill-over effect has been reported by the DHMT and may not be attributed entirely to the pilot.

### Successful Policy Advocacy

Following the pilot project, we continued with advocacy efforts for scale up at the national level. Advocacy efforts included development of an advocacy brief summarizing findings of the pilot as well as the “Spitfire Strategies” advocacy approach introduced by the Gates Population Leadership group (led by Advance Family Planning [AFP] through Jhpiego) for MOH decision-makers. Advocacy messages were mainly aimed at creating a positive policy environment that favored allowing CHWs to provide injectable contraceptives.

In November 2012, the Kenyan government issued a new policy allowing trained CHWs to provide DMPA in hard-to-reach areas.

To date, key medical associations including the Nursing Council of Kenya (NCK), the Midwives Chapter, and the National Nurses Association of Kenya have become supportive of the practice, and NCK has recommended initial scale up to 10 sites/counties where emphasis will be placed on standardization of training, regulation, and supervision. In addition to the key medical associations, we also reached key MOH personnel including the Chief Nurse Officer and the Director of Medical Services, who support the practice because of its approach to improving access for the hard-to-reach populations. Largely as a result of these efforts, the MOH in November 2012 issued an official policy statement allowing provision of DMPA by trained CHWs in hard-to-reach areas.
